# Effects of Heatwaves and Tropical Nights on Sleep in Middle-Aged and Older Adults: A Scoping Review

**DOI:** 10.3390/clockssleep8030037

**Published:** 2026-06-23

**Authors:** Jelena Krčum, Neriman Ezgin, Nikola Šutulović, Nemanja Rajković, Emilija Djurić, Dušan Mladenović, Milena Vesković, Arif E. Cetin, Aleksandra Rašić-Marković, Olivera Stanojlović, Dragan Hrnčić

**Affiliations:** 1 Faculty of Medicine, University of Belgrade, 11000 Belgrade, Serbia; 2 Institute of Medical Physiology “Richard Burian”, Faculty of Medicine, University of Belgrade, 11000 Belgrade, Serbia; 3 Department of Biotechnology, Institute of Natural and Applied Sciences, Cukurova University, Adana 01330, Turkey; 4 Institute of Biophysics, Faculty of Medicine, University of Belgrade, 11000 Belgrade, Serbia; 5 Institute of Pathophysiology “Ljubodrag Buba Mihailovic”, Faculty of Medicine, University of Belgrade, 11000 Belgrade, Serbia; 6 Izmir Biomedicine and Genome Center, Izmir 35340, Turkey; 7 Department of Biophysics, Faculty of Medicine, Dokuz Eylul University, Izmir 35340, Turkey; 8 Department of Electrical and Electronics Engineering, Istanbul Atlas University, Kagithane, Istanbul 34408, Turkey

**Keywords:** heatwaves, tropical nights, sleep quality, middle-aged adults, older adults, thermoregulation

## Abstract

Heatwaves and tropical nights are emerging as significant public health challenges under accelerating climate change, with middle-aged and older adults demonstrating heightened vulnerability. This scoping review maps the existing evidence on how nocturnal heat affects sleep in middle-aged and older adults aged 45 and above, synthesizing findings from experimental and observational studies published in English over the past decade. A comprehensive search of PubMed and Scopus, supplemented by reference screening, identified 31 relevant studies. Data on study design, population characteristics, heat exposure metrics, sleep outcomes, and interventions were charted and synthesized narratively due to methodological heterogeneity. Across studies, elevated nighttime temperatures consistently reduced total sleep time and sleep efficiency, increased wake after sleep onset, and disrupted sleep architecture, particularly REM and N3 stages. Environmental, behavioral, and physiological interventions such as improved ventilation, targeted cooling strategies, and pre-sleep thermal management partially mitigated heat-related sleep disruption. Overall, the findings highlight gaps in standardized exposure metrics and harmonized sleep assessment, providing guidance for future research and public health strategies aimed at protecting sleep health in middle-aged and aging populations amid increasingly frequent extreme heat events.

## 1. Introduction

Heatwaves are increasingly recognized as a major climate-related threat to human health, driven by ongoing global climate change. While acute consequences such as heat stroke, cardiovascular stress, and increased mortality are well-established, less attention has been given to more subtle but widespread health impacts. Among these, the effects of heat exposure on sleep quality have emerged as an important public health concern [1]. In particular, tropical nights, defined as nights in which minimum temperatures remain above approximately 20–25 °C, prevent adequate nocturnal cooling and interfere with the physiological decline in core body temperature necessary for sleep initiation and maintenance [2]. The frequency and intensity of tropical nights have increased in recent decades as a consequence of climate change, contributing to ongoing nocturnal heat stress [3].

Sleep is a vital physiological process, closely dependent on proper body thermoregulation. Sleep onset and stable sleep architecture are dependent on a decline in core body temperature in the evening and during early sleep. However, elevated nighttime temperatures can interfere with this process by limiting heat dissipation through the skin and disrupting circadian regulation [4,5]. Evidence suggests that nocturnal heat exposure can delay sleep onset, fragment sleep, increase awakenings, and reduce overall sleep duration [6]. Large-scale studies further support these findings; for example, analysis of over 23 million nights of sleep indicated that a 10 °C increase in ambient temperature increased the likelihood of insufficient sleep by 20.1% and reduced average sleep duration by nearly 10 min. Despite these findings, the extent of evidence regarding heat-induced sleep disruption across different populations and contexts has not yet been comprehensively mapped.

Middle-aged and older adults appear to be particularly vulnerable to heat stress, as aging is associated with reductions in skin blood flow, sweating efficiency, and overall heat dissipation [7,8]. Epidemiological research shows that higher nighttime temperatures are linked to lower sleep efficiency, prolonged sleep latency, and more frequent awakenings in older populations. Chronic sleep disruption in this group may further exacerbate metabolic, cardiovascular, and cognitive dysfunction and has been associated with accelerated biological and epigenetic aging [9,10,11,12,13]. A more rapid decrease in sleep quality has been documented in women older than 50 years compared to the male population [14].

Given the increasing frequency of extreme heat events and the particular vulnerability of certain populations, there is a need to systematically map the existing evidence on the relationship between heatwaves and sleep. While several primary studies address aspects of this relationship, no comprehensive synthesis exists that captures the range of populations, environmental contexts, and outcomes studied to date. Therefore, the present scoping review aims to provide an overview of current research on the effects of heatwaves and elevated nighttime temperatures on sleep quality with a focus on middle-aged and older adults as particularly vulnerable populations.

## 2. Materials and Methods

### 2.1. Protocol and Registration

This scoping review was conducted according to a predefined protocol designed to map the literature on heatwaves, tropical nights, and sleep outcomes in middle-aged and older adults. The protocol was preregistered on the Open Science Framework (OSF, Registration DOI: 10.17605/OSF.IO/67C4V). Methods followed PRISMA-ScR guidelines to ensure transparency and reproducibility.

#### 2.1.1. Eligibility Criteria

Studies were included based on the following criteria:

Population: Middle-aged and older adults aged ≥45 years exposed to heatwaves or extreme nocturnal heat. Studies included focus on sleep outcomes, thermoregulatory responses, and health effects directly observed in humans. Studies involving animals or in vitro models were not considered. When studies included mixed-age populations, analyses were restricted to results reported for middle-aged and older adults aged 45 years and older. If age-stratified outcomes were available, only the relevant subgroup data were analyzed. Studies employing age thresholds of ≥45 years were considered eligible when age-stratified data for middle-aged and older adults were available and clearly reported.

Exposure: Documented heatwave events, extreme ambient temperatures, short-term nocturnal exposure, or prolonged multi-night exposure. Definitions of these events have been retaken from the included studies, assessed and reported as they are, while analyzed with caution to respect the definition of tropical nights as nights in which minimum temperatures remain above approximately 20 °C.

Comparators: Cooler ambient conditions, pre-heatwave periods, seasonal averages, or controlled laboratory conditions.

Outcomes:

Primary: Sleep quality measured subjectively (PSQI, ISI, sleep diaries) or objectively (polysomnography, actigraphy, wearable sensors).

Secondary: Sleep continuity, latency, efficiency, fragmentation, architecture, circadian rhythm parameters, next-day sleepiness or fatigue.

Study Type: Original research articles (observational, experimental, interventional).

- Publication Date: January 2015–February 2026.
- Language: English.

#### 2.1.2. Exclusion Criteria

- Letters, case reports or review articles.

#### 2.1.3. Information Sources

A comprehensive literature search was performed in PubMed/MEDLINE and Scopus for studies published from January 2015 to February 2026. Manual reference screening of included articles was conducted to identify additional relevant studies. The most recent search was executed on 10 February 2026.

#### 2.1.4. Search Strategy

A structured search strategy combined MeSH terms and keywords using Boolean operators:

(“heatwave” OR “extreme heat” OR “high temperature” OR “tropical night” OR “heat stress”) AND (sleep OR “sleep quality” OR “sleep disturbance” OR “sleep architecture” OR REM OR N3 OR “sleep efficiency” OR “wake after sleep onset” OR WASO OR “short sleep” OR “sleep duration” OR insomnia OR “sleep apnea” OR “sleep apnoea” OR sleep-disordered breathing”) AND (“aging” OR “elderly” OR “older”)

Search limits: English language, human subjects.

#### 2.1.5. Selection of Sources of Evidence

Study selection was conducted in two stages:

1. Title and abstract screening to identify potentially relevant studies.
2. Full-text review to confirm eligibility.

Screening was performed independently by two reviewers, with disagreements resolved through discussion. The process is documented in a PRISMA flow diagram.

#### 2.1.6. Data Charting Process

Data extraction was performed using a pretested charting form capturing study characteristics, population demographics, heat exposure definitions, sleep assessment methods, interventions, and main findings. Extraction was conducted independently by two reviewers and cross-checked for consistency. Additional data were requested from study authors when necessary.

#### 2.1.7. Data Items

##### Variables Extracted Include

The variables extracted from the included studies encompassed study design and country, sample size and participant age, type and definition of heat exposure, sleep outcomes and their measurement methods, and interventions, as well as key results and effect sizes.

Assumptions were clearly noted where data were incomplete.

##### Critical Appraisal of Individual Sources of Evidence

Consistent with scoping review methodology, formal risk-of-bias scoring was not performed. Instead, methodological rigor, sample size, exposure assessment accuracy, and outcome measurement reliability were evaluated qualitatively by two independent reviewers, with discrepancies resolved through discussion, to contextualize findings.

#### 2.1.8. Data Synthesis and Quality Context

Given the heterogeneity in study designs, heat exposure definitions, and sleep outcome measures, a qualitative synthesis was conducted. Data from all included studies were charted and summarized narratively to identify patterns, trends, and gaps in the evidence regarding the effects of heatwaves and tropical nights on sleep in middle-aged and older adults. Key outcomes synthesized included changes in sleep quality, sleep architecture, continuity, and efficiency.

Although no formal scoring system was applied, the dual-reviewer evaluation ensured systematic assessment of methodological quality and interpretation of findings.

## 3. Results and Discussion

### 3.1. Results

**Figure 1:**
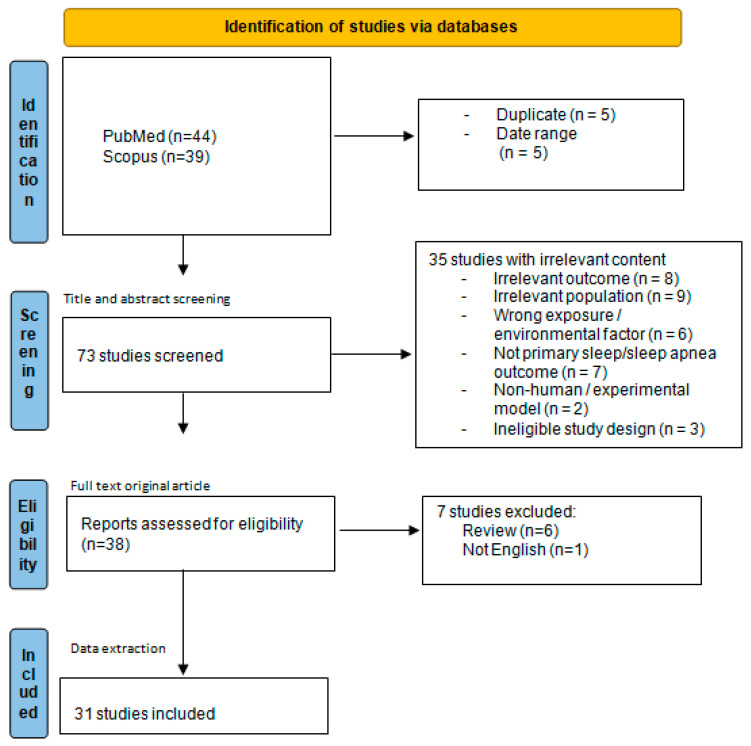
PRISMA flow diagram.

A total of 83 records were identified through database searching, including PubMed (n = 44) and Scopus (n = 39). During the title and abstract screening phase, 10 records were excluded due to date range and duplication, leaving 73 studies for further assessment. Subsequently, 35 studies were excluded according to predefined eligibility criteria, including irrelevant outcome, irrelevant population, inappropriate exposure, ineligible study design, and lack of outcome specificity. As a result, 38 full-text articles were assessed for eligibility. Of these, seven studies were excluded. Ultimately, 31 studies were included in the final analysis, and data extraction was performed on these studies. The study selection process is summarized in Figure 1 (PRISMA flow diagram).

Key characteristics of each study, including author, year, country/region, cohort/population details, sample size and age distribution, heat exposure type and duration, comparator groups, sleep outcomes, main findings, and methodological quality or risk of bias, are summarized in Table 1.

#### 3.1.1. Study Design and Populations

The 31 studies included in this scoping review investigated the effects of ambient temperature, heatwave exposure, and seasonal variation on sleep among adult and older populations. Study designs were heterogeneous, encompassing longitudinal cohort studies, repeated-measure and fixed-effect observational designs, prospective seasonal field studies, cross-sectional surveys, laboratory experiments, community-based interventions, mixed-method case studies, time-use surveys, and large-scale multinational panel analyses and prospective device-based OSA monitoring studies [15,16,17,18,19,20,21,22,23,24,25,26,27,28,29,30,31,32,33,34,35,36,37,38,39,40,41,42,43,44,45].

Sample sizes varied widely, ranging from intensive small-scale monitoring studies with fewer than 20 participants to very large-scale national and multinational datasets comprising hundreds of thousands to millions of sleep observations derived from wearable devices and population-based cohorts [25,26,27,28,29,30,31,32]. The largest samples in this review were contributed by OSA-focused wearable studies, with Li et al. (2024) including 51,842 participants across 313 Chinese cities totaling 6,232,056 monitoring days [33]. Intermediate-sized studies typically included several hundred to tens of thousands of participants, often employing longitudinal or repeated-measure designs to strengthen within-subject inference [34,35,36,37].

**Table 1:** Characteristics of included studies.

Author(s), Year	Country/ Region	Study Design/ Intervention	Population Characteristics	Sample Size (n)	Heat Exposure Definition/Duration	Comparator /Control	Sleep Outcomes /Measures	Key Findings
Ding et al., 2025 [15]	USA (Boston, MA)	Repeated-measure home-based monitoring	Older adults, 67.7% female, 90.3% White, 12 cognitively impaired	62	≥3 consecutive nights of sleep monitoring	Consecutive nights	SleepImage Ring: TST, SE, latency, WASO, REM, deep/light sleep, HR, SpO_2_	Seven sleep metrics were relatively stable across nights, while sleep latency showed the greatest variability
Zhou et al., 2025 [16]	China, 23 provinces	Longitudinal cohort (CLHLS 2008–2018)	≥65 yrs, mean 84.2, 51.2% female	9153	Heatwave: ≥3 days above 92.5th percentile	Long sleep + heatwave as reference	Self-reported sleep duration/quality	Combined exposure to heatwaves and long sleep duration increased the risk of cognitive impairment in older adults.
Zhou et al., 2024 [17]	China	Longitudinal cohort (CHARLS)	≥60 yrs, mean 67.5, 50.2% female	7240	City-level heatwave: ≥2–4 days above 90–97.5th percentile	Non-heatwave periods	Self-reported sleep duration	Heatwave exposure reduced sleep duration, stronger in women/urban/chronic disease
Huang et al., 2022 [36]	China, Jiangsu	Intervention study	Rural elderly, 57.9–60.1 yrs	41	90th percentile daily max temp 32 °C	Control vs. education/subsidy/cooling	Wearable smart band: TST and sleep stage indicators	Interventions mitigated sleep reduction, improved DSD/TST
Kume et al., 2017 [18]	Japan & Thailand	Prospective seasonal	Community-dwelling older adults in Japan and Thailand	Japan 37, Thailand 44	Seasonal variation, humid subarctic & tropical savanna	Across seasons/country	Actiwatch 2: TST, latency, SE, awakenings, rest–activity rhythm	Seasonal effects on TST and rhythm; Japanese summer shortest sleep, Thai poor year-round
van Loenhout et al., 2016 [19]	Netherlands	Prospective, observational	Community-dwelling ≥ 65 yrs, 51% male	113	Indoor temp > 25 °C during summer weeks	Cold reference week	Hourly diary: sleep disturbance	Bedroom temp predicted sleep disturbance; thirst, sweating also high
Williams et al., 2019 [20]	USA, Cambridge, MA	Observational multi-day	Low-income older adults ≥ 55 yrs	51	Extreme heat event summer 2015, indoor temp means non-AC 25.6 °C	Central AC vs. non-AC	Actigraphy, HR, GSR, self-reported sleep	Higher indoor temp → more sleep disturbance, HR & GSR ↑
Hajdu, 2024 [21]	Hungary	Time-use survey, longitudinal	Adults 18–84 yrs, 61+ 15–20%; subgroup analyses performed	46,446	Daily mean temp; heatwave ≥ 3 or ≥5 days > 25 °C	5–10 °C reference	Sleep diaries: TST, bed/wake times, binary < 6 h	Hot days reduced sleep 5–25 min; stronger in older adults/men
Tang et al., 2025 [22]	China	CHARLS cohort	≥45 yrs, median 57	9475	Heatwave ≥ 92.5–97.5 percentile, cold ≤ 2.5–7.5 percentile, ≥2–4 days	Lower exposure	Self-reported sleep duration (<6 h)	Short sleep duration increased susceptibility to temperature-related multimorbidity among older adults
Cepeda et al., 2018 [23]	Netherlands, Rotterdam	Prospective cohort (Rotterdam Study)	≥50 yrs, 34% 50–64, 39% 65–74, 28% ≥75	1166	Daily avg temp, sunlight, humidity, wind, precipitation	Within-participant seasonal comparison	Actigraphy + sleep diary: TST, time in bed, SE	Nighttime TST highest mid-Jan; elderly no seasonality; temp explained 49.4% of variation in middle-aged
Cheong & Gaynanova, 2024 [24]	USA, Houston, TX	Community-based, 2-week measurement	Mean 58.2 yrs, 70% female, 76.7% African American	30	Person-specific ambient temp 25–38 °C, wrist accelerometer	Frequent vs. infrequent AC	TST, SE, sedentary time	1 °C ↑ → SE ↓ 2%, sedentary ↑ 2%; AC use mitigated effects
Uriarte-Otazua et al., 2025 [26]	Spain, Basque Country	Mixed-method case study	72 & 78 yrs, living alone	2	Indoor night temps up to 28.7 °C, daytime up to 30 °C	Cooler nights/peri-urban vs. urban	Interviews, thermal monitoring, inferred sleep	Urban dwelling → poor sleep; peri-urban better due to ventilation/mobility
Son et al., 2024 [27]	USA, Phoenix, AZ	Cross-sectional, 3 days	≥54 yrs, independent seniors	18	Nighttime bedroom temperature, light exposure	Within-subject	Actigraphy, light tracker: TST, SE, fragmentation	Higher nighttime bedroom temperature → reduced sleep fragmentation, improved sleep efficiency, negative correlation with sleep duration; greater daytime light exposure → longer total sleep time
Yan et al., 2022 [28]	Shanghai, China	Laboratory experiment	≥65 yrs	16	27 °C vs. 30 °C, ventilation vs. no ventilation	27 °C baseline	TST, SE, REM, deep sleep, time awake (PSG, physiological measures)	30 °C → TST −26.3 min, SE −5.5%, REM −5.3 min, time awake +27 min; ventilation → deep +10.3 min, REM +3.7 min
Baniassadi et al., 2023 [37]	USA (Boston metropolitan area)	Prospective longitudinal observational home-based study (12-month repeated-measure; GAM + causal modeling)	Community-dwelling adults ≥ 65 yrs; cognitively intact; independently living; relatively high SES; non-dementia	50 (10,903 person-nights)	Nighttime bedroom temperature continuously measured (Netatmo sensors; ~14–32 °C; 15 min intervals; 12 months)	No external control group; within-subject variation (each participant serves as own control)	TST, SE, RSTLS (Oura ring wearable; validated against polysomnography)	Non-linear relationship; optimal sleep at 20–25 °C; >25 °C associated with ↓SE (≈5–10%), ↓TST (~60 min less at 30 °C vs. 22 °C), ↑restlessness; strong inter-individual variability
Minor et al., 2022 [29]	Global (68 countries; multi-continental dataset)	Large-scale observational panel study using accelerometry-based sleep tracking; fixed-effect econometric models; climate–sleep linkage analysis (2015–2017)	Adult population (broad global sample; heterogeneous by age, sex, income; includes elderly sub-analyses); users of sleep tracking wearable devices	7.41 million sleep records (10+ billion sleep observations; repeated daily measures across 68 countries)	Nighttime outdoor minimum temperature matched to geolocated sleep data (meteorological station + gridded climate data; continuous exposure)	Within-individual fixed-effect design (each person serves as their own control; comparison across temperature variation within individuals and regions)	Total sleep time (TST), sleep timing (sleep onset, midsleep, offset), probability of short sleep (<7 h, <6 h, <5 h) from accelerometry-based wristband data	Higher nighttime temperatures reduce sleep duration (~14 min loss > 30 °C), delay sleep onset, advance wake time, and increase probability of short sleep; strongest effects in elderly, females, and low-income regions; no clear adaptation over time; projected climate change may increase annual sleep loss (~44 h/year historically, increasing to ~50–58 h by 2099 under RCP scenarios)
Liao J et al., 2025 [30]	United States (All of Us Research Program; nationwide cohort)	Longitudinal observational cohort study; multilevel mixed-effect panel models; climate–sleep linkage + CMIP6 projections	Adults ≥ 18 yrs; predominantly female and White; heterogeneous SES; Fitbit users linked with EHR and survey data; subgroup analyses performed	14,232 participants, of which 7689 older than 50 years (12.5 million sleep tracking days, of which 7,317,214 for 50+ subgroups)	Daytime (DTA) and nighttime (NTA) temperature anomalies derived from gridMET (~4 km resolution); ZIP-code-linked exposure over 1990–2023 climatology	Within-person longitudinal comparison using mixed-effect models (participant-level random effects; adjustment for time-varying meteorology and fixed covariates)	Total sleep time, sleep efficiency (SE), wake after sleep onset (WASO), sleep onset timing, REM/deep/light sleep (Fitbit wearable data)	Higher nighttime temperature reduces total sleep time (~2.68 min per 10 °C NTA for age group 50–65 and ~2.39 min per 10 °C NTA for age 65+), worsens sleep efficiency, increases WASO, delays sleep onset, and reduces REM/deep/light sleep. Population aged 40–50 years showed decreased total sleep duration 19.6% higher than population less than 40. The older age population (65 yrs+) did not show a significant difference compared to the youngest age group (<40 years)
Li, A. et al., 2025 [31]	China (mainland; 336 cities at prefecture level or above)	Nationwide repeated-measure observational study; generalized linear models + linear mixed-effect models; fixed-effect sensitivity analyses; climate projection modeling using SSP scenarios	Adults ≥ 18 years using Huawei wearable devices; mean age 39.2 ± 12.8 years; 36.0% BMI ≥ 25 kg/m^2^; subgroup analyses performed for age strata younger and older than 45 years (N.B. limitations for interpretation, it included 45–50 years population also)	214,445 participants; 23,197,045 sleep monitoring days; 30% random sample of eligible users	24 h mean ambient temperature (ERA5-Land reanalysis, 0.1° resolution) linked to residential location; lag 0 (24 h before awakening); 2021–2023	Within-individual repeated-measure (random intercept per participant; no external control group)	Sleep insufficiency (<7 h), total sleep duration, light sleep, deep sleep, dream sleep; measured via Huawei wearable (PPG + accelerometer-based sleep staging algorithm)	In age group age ≥ 45, each 10 °C increase associated with +23.0% odds of sleep insufficiency; −10.92 min total sleep; −5.04 min light sleep; −3.61 min deep sleep; −2.31 min dream sleep. The associations of high temperature with sleep insufficiency and sleep duration were consistently stronger in age group ≥ 45 years, women, those with a BMI ≥ 25 kg·m^−2^, and those simultaneously experiencing high humidity
Basner et al., 2023 [34]	USA (Louisville, Kentucky)	Observational longitudinal field study (14-day real-world monitoring with actigraphy + bedroom environmental sensors)	Community-based adult sample (GHP subgroup); age range 25–70 years; individuals with diagnosed sleep disorders excluded	62	Continuous bedroom environmental temperature exposure (also PM2.5, CO_2_, humidity, noise); high-frequency (1 min) measurements across 14 days	Exposure stratified into quintiles (lowest vs. highest exposure categories); within-subject and mixed-effect modeling	Actigraphy-based sleep efficiency (primary outcome), total sleep time (TST), wake after sleep onset (WASO), sleep onset latency; subjective sleep quality and sleepiness	Higher bedroom temperature associated with significantly lower sleep efficiency (≈3.4% reduction in highest vs. lowest quintile). Effects also observed for PM2.5, CO_2_, and noise. No strong consistent association with total sleep time. Effects appear additive across environmental stressors
Guo et al., 2023 [38]	Tibet Autonomous Region, China	Seasonal observational field study (winter vs. summer), 1-week real-world monitoring	Healthy high-altitude adults (≥40 years included within 26–64 age group); no chronic disease; non-smokers; long-term residents	Total: 197 participants (97 summer, 100 winter)	Bedroom microclimate exposure measured continuously for 1 week: temperature (Ta), relative humidity (RH), CO_2_ (2 min intervals)	Winter vs. summer comparison (between different participant groups; no separate ≥40 subgroup analysis)	Fitbit-based sleep metrics: TST, SE%, WASO, REM, Light, Deep, NOA + GSQS + AHS symptoms	In adults including ≥40 group: sleep becomes more fragmented (↑WASO, ↑NOA), lower deep and REM sleep, higher light sleep. In summer, higher CO_2_, temperature, and humidity significantly worsen sleep efficiency and deep sleep; CO_2_ is the strongest predictor. In winter, low humidity (dry air) is the main factor reducing sleep duration and increasing discomfort. Older adults are more sensitive to environmental stressors affecting sleep quality
Hailemariam et al., 2023 [35]	Australia	Longitudinal panel data analysis (HILDA 2001–2019), fixed-effect regression + temperature bins	National adult population (household survey; includes adults across all ages, including ≥40 subgroup not separately isolated)	97,959 observations (≈individuals repeated over waves 1–19)	Daily mean/max/min temperature; binned exposure (<40 °F, 40–50 °F, … >90 °F); monthly aggregation	Reference category: 60–70 °F; within-individual fixed-effect comparison	Self-reported sleep quality (4-point scale; only waves 13 & 17)	Overall findings: inverted U-shaped effect on general health; hot and cold days reduce health; weak/no robust effect on subjective wellbeing; sleep quality does not mediate temperature–health link
Lappharat et al., 2018 [39]	Thailand (Bangkok)	Cross-sectional observational study	Adults with diagnosed obstructive sleep apnea (OSA); median age 42 years; 73% male; mean BMI ≈ 26.2	63	Bedroom temperature measured continuously during sleep using data loggers; exposure assessed over 3 consecutive nights in wet and dry seasons (1-year mean calculated)	No explicit control group; comparisons based on variations in environmental exposure (temperature, PM10, humidity)	Polysomnography (AHI, RDI), Pittsburgh Sleep Quality Index (PSQI), sleep latency, duration, efficiency	Higher bedroom temperature associated with poorer subjective sleep quality (OR 1.46, *p* = 0.044); PM10 associated with increased OSA severity (AHI, RDI); temperature not significantly associated with AHI
Li W. et al., 2020 [40]	USA (Greater Boston Area, Massachusetts)	Prospective cohort study with repeated daily measures (actigraphy-based sleep + daily environmental exposure assessment); fixed-effect statistical models	Adults with episodic migraine; predominantly women (88%); mean age ~35 years; generally healthy, no severe sleep apnea or major comorbid exclusions	98 participants (4406 total sleep nights)	Daily ambient temperature (°F/°C), relative humidity, barometric pressure; exposure assessed daily over ~6 weeks (average follow-up 45 days per participant)	Within-person comparison (each participant serves as their own control across different days); fixed-effect models controlling for day-of-week and seasonal trends	Objective actigraphy: total sleep duration, wake after sleep onset (WASO), sleep efficiency; self-reported sleep latency and sleep quality (daily diary)	Higher temperature → ↑ WASO and ↓ sleep efficiency (modest effects). Higher ozone (O_3_) → longer sleep duration. No consistent associations for PM2.5 or most pollutants. No strong seasonal differences
Liu et al., 2022 [41]	Taiwan	Cross-sectional hospital-based observational study (retrospective PSG + environmental exposure modeling)	Mean 49.5 ± 13.5 years predominantly middle-aged OSA patients	5204	Ambient temperature and relative humidity estimated using 1-day, 7-day, 1-month, 6-month, 1-year averages from monitoring stations	Internal comparison across different exposure levels (low vs. high RH/temperature over time)	PSG parameters: AHI, sleep efficiency, WASO, arousal index, sleep stages (N1–REM), oxygen desaturation	Higher long-term temperature → lower sleep efficiency and higher WASO; higher RH → mixed effects (↑AHI, altered sleep stages, ↑arousals); humidity had stronger effect on sleep architecture and oxygen desaturation than temperature
Milando et al., 2022 [42]	ABD (Boston, Massachusetts; Chelsea & East Boston)	Mixed-method observational field study using wearable sensors, environmental monitors, and qualitative interviews	Urban residents in low-income, environmentally overburdened communities; predominantly female, Hispanic/Latino, renters in multi-family housing	24 participants (22 with complete sensor data)	Summer 2020 (6–8 weeks); personal, indoor, and outdoor temperature measured using wearable and fixed sensors	Natural comparison between personal, indoor, and outdoor (weather station) temperature measurements	Sleep duration measured via Fitbit wearable device during nights when participants were at home	Personal heat exposure was ~3.9 °C higher than weather station temperatures; air conditioning did not fully control indoor heat; no statistically significant association between indoor temperature and sleep duration; substantial heterogeneity in heat-related behaviors
Obradovich N et al., 2019 [43]	United States	Pooled cross-sectional econometric analysis (fixed-effect regression using BRFSS 2002–2011 + meteorological linkage)	General adult population (BRFSS respondents); subgroup analyses performed for age ≥ 65, income levels	766,761 (main sample for sleep outcome analysis)	30-day average nighttime temperature anomalies (deviation from 1981 to 2010 normals)	Relative comparison across temperature anomaly levels (fixed-effect adjusted within-city and time variation)	Self-reported insufficient sleep (BRFSS question: number of days with insufficient sleep in past 30 days)	Higher nighttime temperature anomalies significantly increased insufficient sleep (β ≈ 0.028, *p* = 0.014). Stronger effects in older adults (≥65: β ≈ 0.041) and low-income groups. Summer effects strongest
Tsuzuki K [44]	Japan (Nagoya, 35.17° N, 136.77° E)	Observational longitudinal field study; repeated measures across four seasons (spring, summer, fall, winter); no intervention (naturalistic environmental exposure)	Older adults living independently in public elderly care facilities; screened for chronic disease, insomnia, medication use, hospitalization history, and dementia	16 initially recruited (8 men, 8 women); 13 participants completed all four seasons (5 men, 8 women)	Continuous indoor environmental exposure (air temperature and relative humidity) measured in bedrooms over 1 week per season (4 seasons); seasonal variation in ambient heat (summer up to ~29 °C at night)	Within-subject seasonal comparisons (spring vs. summer vs. fall vs. winter); sex-based comparison (men vs. women); no external control group	Actigraphy (sleep–wake cycles, sleep efficiency index, total sleep time, sleep latency, wake after sleep onset, activity index); sleep diaries; subjective sleep questionnaires; thermal sensation/comfort scales	Summer indoor temperatures exceeded 28–29 °C at night; elderly men showed worse sleep efficiency and longer wake time than women; sleep parameters showed limited seasonal significance but clear sex differences; higher temperatures associated with altered thermal sensation (often negative correlation with expected direction); adaptive behaviors (fans/open windows) partly used; thermal comfort generally aligned with adaptive comfort model
Wang C et al., 2022 [32]	China (162 counties, 25 provinces)	Observational longitudinal study using nationally representative survey data combined with county-level meteorological data; fixed-effect regression (individual, county, time)	Adults ≥ 18 years (nationally representative). Subgroup analyses performed for elderly (≥60 years), rural/urban residents, gender, and education levels	~30,000 adults per wave (2012 & 2016); total ~45,319 individuals in CFPS waves (sleep module subset used in analysis)	County-level daily temperature matched to respondents over past 7-day recall period. Exposure: average temperature, extreme heat (>28 °C), degree-days (≥16 °C threshold)	Reference temperature: 16–20 °C (optimal sleep range). Within-individual comparison using fixed effects (no separate external control group)	Self-reported sleep experience (CES-D scale item): restless sleep frequency over past week; binary outcome = “poor sleep” (5–7 restless days)	≥60 subgroup finding: Elderly individuals show stronger sensitivity to high temperature, with significantly higher likelihood of poor sleep compared to younger groups. Heat effects are amplified due to reduced thermoregulation capacity. Overall: heat increases poor sleep risk, especially in rural, low-education, and elderly populations
Weinreich G et al., 2015 [45]	Germany (Ruhr area: Essen, Bochum, Mülheim)	Population-based prospective cohort study	Adults aged 45–75 at baseline, follow-up screening at 50–80 years (includes older/elderly subgroup ~60+)	1773 participants (final analytic sample)	Daily mean temperature (lag 0, 0–2 days moving averages), derived from local meteorological stations; exposure during screening night and short-term periods	Lower temperature days/interquartile range comparison	Sleep-disordered breathing measured via apnea–hypopnea index (AHI) using ApneaLink device	Higher temperature associated with increased AHI; e.g., IQR increase in temperature linked to ~10% increase in AHI; stronger effects in summer
Li et al., 2024 [33]	China (313 cities, mainland)	Prospective observational; wearable PPG-based smartwatch monitoring + DLNM statistical model	Adults with moderate-to-severe OSA risk; mean age 45.4 yrs (SD = 11.0); 95.5% male; 69.1% BMI ≥ 24. Note: Age-stratified data reported for ≥45 yrs only; ≥50 yrs subgroup not separately available	n = 51,842 (6,232,056 monitoring days)	24 h mean ambient temperature (lag 0 d); continuous variable; range −5.2 °C to 31.4 °C; extreme high = 30.3 °C (97.5th percentile)	Referent = minimum temperature (−5.2 °C); within-subject comparison across nights	OSA exacerbation (AHI ≥ 5/h, binary); AHI (events/h); MinSpO_2_ (%)	In the ≥45 yrs subgroup, high temperature was associated with significantly greater OSA exacerbation compared to <45 yrs (*p* < 0.05); subgroup-specific numeric estimates not reported. Overall cohort per 10 °C↑: OSA exacerbation OR +8.4% (95% CI 7.6–9.3%); AHI +0.70 events/h (95% CI 0.65–0.76); MinSpO_2_ −0.18% (95% CI −0.19 to −0.16). Effects present on same day only (lag 0); disappear at lag 1+
Lechat et al., 2025 [25]	Global (41 countries; 29 with significant associations)	Prospective longitudinal observational; FDA-cleared under-mattress nearable sensor (Withings Sleep Analyzer); 3.5-year multi-night monitoring + CMIP6 climate burden modeling	Adults ≥ 18 yrs with OSA; predominantly male (77.3%); middle-aged; median 509 nights/user. Age subgroup analyses performed in 10-year categories; age did not strongly modify the temperature–OSA association	n = 116,620 (~62 million nights)	24 h average ambient temperature (ERA5); extreme high = 99th percentile (27.3 °C); 4-day lag; real-world nightly exposure	25th percentile temperature (6.4 °C)	Nightly OSA status (AHI ≥ 15 events/h); nightly severe OSA (AHI ≥ 30 events/h)	High temperatures (99th vs. 25th percentile) associated with 45% higher probability of nightly OSA (RR 1.45, 95% CI 1.44–1.47) and 49% higher for severe OSA (RR 1.49, 95% CI 1.46–1.52). Age did not strongly modify the association. Effect stronger in males and higher BMI. European countries showed strongest effects (~2-fold). Warming-related OSA burden 2023: ~788,198 DALYs lost; $30 billion productivity loss across 29 countries. Scenarios ≥ 1.8 °C above pre-industrial levels projected to double OSA burden by 2100

Most participants were community-dwelling adults, with a substantial proportion of studies focusing on middle-aged and older adults (≥45–65 years and above), who were consistently identified as particularly vulnerable to heat-related nocturnal stress and sleep disturbances [17,18,19,32]. Adults aged ≥45 years showed significantly stronger temperature-related OSA exacerbation compared to younger adults [33] though this age-modifying effect was not consistently observed across studies, as Lechat et al. (2025) found that age did not strongly modify the temperature–OSA association across 10-year age categories [25]. However, several studies included heterogeneous adult populations without explicit age-stratified analyses, limiting direct inference for older adult-specific outcomes [35,42].

#### 3.1.2. Geographical Regions Included

Studies were conducted across North America, Europe, Asia, Oceania, and global multi-country settings, reflecting diverse climatic, geographic, and socioeconomic contexts. In particular, studies were performed in the United States of America [15,20,24,29,30,37,40,42,43], the Netherlands [19,23], Spain [26], Hungary [21], Germany [33], China [16,17,22,28,31,32,33,36,37,39,43], Japan [18], Thailand [18,41], Taiwan [43], and Australia [34], as well as global multi-country cohorts spanning 41 countries [25] and 68 countries [29].

#### 3.1.3. Measurements of Sleep Outcomes

Sleep outcomes were assessed using both objective and subjective methods across studies, including wearable technologies, polysomnography, actigraphy, under-mattress sensors, and self-reported instruments such as sleep diaries and questionnaires.

Subjective sleep outcomes commonly included self-reported total sleep duration, sleep quality, sleep satisfaction, ease of sleep initiation, and nocturnal awakenings. For example, Huang et al. [36] assessed self-reported sleep duration and quality in response to heat-relief interventions, while Williams et al. [20] recorded nighttime awakenings and perceived sleep disruption during elevated indoor temperatures. Van Loenhout et al. [19] evaluated perceived sleep disturbance under high bedroom temperatures, and Hajdu [21] derived sleep duration and sleep timing from time-use diaries. Similarly, Zhou et al. [16] and Tang et al. [22] used self-reported sleep duration and short sleep indicators in large cohort studies, while Wang et al. [32] assessed sleep quality and insufficient sleep using survey-based measures. Lappharat et al. [39] additionally used the Pittsburgh Sleep Quality Index (PSQI) alongside clinical sleep outcomes.

Objective sleep measurements were widely obtained using wearable devices and sensor-based technologies. Ding et al. [15] used smart wearable rings to measure total sleep time (TST), sleep efficiency (SE), sleep onset latency, wake after sleep onset (WASO), REM and deep sleep, sleep fragmentation, heart rate, and oxygen saturation. Zhou et al. [17] and Zhou et al. [16] applied actigraphy to assess TST, SE, sleep latency, WASO, and sleep stage distribution. Lechat et al. [25] conducted large-scale monitoring using under-mattress sensors and wearable devices across 41 countries, capturing TST, sleep onset, awakenings, and short sleep prevalence. Minor et al. [29] used global accelerometry-based wearable data to derive TST, sleep timing, and short sleep probability across 68 countries. Li et al. (2024) [33] employed PPG-based smartwatch monitoring to assess OSA exacerbation (AHI ≥ 5 events/h), apnea–hypopnea index (AHI), and minimum oxygen saturation (MinSpO_2_).

Cheong & Gaynanova [24] and Li W. et al. [44] used wrist-worn accelerometers to assess TST and SE in relation to ambient temperature and air pollution exposure. Baniassadi et al. [37] applied Oura ring data to evaluate TST, SE, and restlessness under varying bedroom temperatures. Liao et al. [30] and Li et al. [31] utilized large-scale wearable datasets to assess sleep parameters such as total sleep time, sleep efficiency, and wake after sleep onset (WASO). Basner et al. [34] combined actigraphy with environmental sensors to assess SE, TST, WASO, and sleep onset latency in relation to bedroom temperature and air quality.

Clinical and laboratory-based studies provided more detailed physiological sleep assessment. Yan et al. [28] used polysomnography (PSG) to measure TST, SE, REM sleep, deep sleep, and wake time under controlled temperature conditions. Hashizaki et al. [40] used contactless biomotion sensors to estimate sleep–wake cycles, sleep efficiency, WASO, and circadian timing across seasons. Lappharat et al. [39] also applied PSG to evaluate apnea–hypopnea index (AHI), respiratory disturbance index, sleep efficiency, and sleep architecture.

Additional field-based and mixed-method studies incorporated environmental and qualitative components. Milando et al. [42] combined Fitbit-derived sleep duration with environmental heat exposure and qualitative interviews. Uriarte-Otazua et al. [26] integrated thermal monitoring with qualitative interviews to infer sleep disturbance under heat exposure. Hailemariam et al. [35] and Obradovich et al. [43] relied on self-reported sleep insufficiency and sleep quality within large population datasets.

Overall, across studies, sleep outcomes consistently included core objective indicators (TST, SE, WASO, sleep latency, sleep stages) and subjective indicators (sleep quality, duration, and disturbance), with measurement approaches ranging from high-resolution physiological monitoring to large-scale survey-based assessments.

#### 3.1.4. Heat Exposure Definitions

Heat exposure definitions varied considerably across studies, reflecting different methodological approaches ranging from population-level meteorological indices to individual-level sensor-based measurements. The most common approach was percentile-based heatwave definitions, typically using thresholds such as ≥90th, ≥92.5th, or ≥97.5th percentiles of temperature sustained for 2–5 consecutive days [16,17,22,25,29,31,44]. Some studies additionally defined extreme heat using absolute temperature thresholds or degree-day models, such as daily mean temperatures above ~25–32 °C or heatwave periods exceeding regional climatological baselines [21,32,37].

Indoor and microclimate-based exposure definitions were also widely used. Several studies defined heat exposure based on indoor bedroom temperature thresholds, particularly values exceeding 25 °C or reaching up to 28–30 °C during nighttime conditions [19,20,26,28,29]. In controlled laboratory settings, experimental heat exposure was defined as fixed ambient temperature conditions (e.g., 27 °C vs. 30 °C) with or without ventilation manipulation [28], allowing precise isolation of thermal effects on sleep architecture.

Several studies incorporated wearable or personal exposure-based measurements, capturing individual-level ambient temperature variations rather than relying solely on external weather data. For example, Cheong & Gaynanova [24] used wrist-based accelerometers combined with personal ambient temperature monitoring (25–38 °C range), while Baniassadi et al. [37] continuously measured bedroom temperature using sensor networks across 12 months (approximately 14–32 °C). Li A. et al. [31] and Liao et al. [30] derived exposure from high-resolution gridded reanalysis data linked to residential location, capturing day-to-day variability in ambient temperature. Similarly, Li et al. (2024) used 24 h mean ambient temperature derived from national meteorological stations matched to participants’ residential districts, covering a continuous range of −5.2 °C to 31.4 °C [33]. Milando et al. [42] further distinguished between personal, indoor, and outdoor temperature exposure, demonstrating discrepancies between perceived and measured heat exposure.

In addition, some studies defined heat exposure using broader environmental or climatic constructs, including tropical or seasonal heat patterns and hot-night conditions. For instance, Kume et al. [18] examined seasonal variation across tropical and subarctic climates, while Guo et al. [38] assessed combined effects of high temperature, humidity, and CO_2_ concentration across seasonal (summer vs. winter) indoor environments. Hailemariam et al. [35] used continuous daily meteorological variables (maximum, minimum, and mean temperature) to model heat exposure without fixed heatwave thresholds.

Overall, the included studies reflect substantial heterogeneity in heat exposure definitions, ranging from percentile-based heatwave indices and absolute temperature thresholds to indoor environmental monitoring and individualized wearable-based exposure assessment. This variability aligns with the exploratory nature of the evidence base and highlights the lack of a standardized definition of heat exposure in sleep research. A qualitative assessment of methodological quality and potential bias, rather than a formal risk-of-bias score, is presented in Table 1. Most studies were observational in design, with frequent reliance on self-reported outcomes or wearable-derived estimates, and many lacked blinding or randomized exposure allocation. Sample sizes varied widely—from very small experimental or case studies to large multinational cohorts—limiting direct comparability and generalizability across findings.

#### 3.1.5. Key Findings

Heat exposure consistently disrupted sleep in middle-aged and older adult populations across observational, experimental, and longitudinal studies. Total sleep time (TST) decreased by approximately 10–45 min per night in heat-exposed conditions [20,24,25,30,31], with some global analyses reporting even larger cumulative losses under sustained high nighttime temperatures [29]. Sleep efficiency (SE) consistently declined by about 5–15% in older or middle-aged cohorts, particularly under elevated indoor temperatures or heatwave conditions [19,31,34,37].

Sleep architecture was also affected. REM and deep (N3) sleep were reduced in controlled and field-based studies, including laboratory heat exposure [28], wearable-based monitoring [15,36], and seasonal field studies [18,38]. Wake after sleep onset (WASO), sleep fragmentation, and nocturnal awakenings increased significantly with higher ambient or indoor temperatures [30,34,40]. Sleep onset latency and circadian misalignment were also reported, particularly in studies examining repeated or seasonal exposure [17,18,23].

Beyond general sleep disruption, heat exposure was also associated with worsening of sleep-disordered breathing. Li et al. (2024) demonstrated a near-linear association between ambient temperature and OSA severity, with each 10 °C increase in daily temperature associated with an 8.4% increase in the odds of OSA exacerbation (95% CI 7.6–9.3%), an increase in AHI of 0.70 events/h (95% CI 0.65–0.76), and a decrease in minimum oxygen saturation of 0.18% (95% CI 0.16–0.19%), with effects present on the same day only [33]. Lechat et al. (2025) further demonstrated that high ambient temperatures (99th vs. 25th percentile) were associated with a 45% higher probability of nightly OSA (RR 1.45, 95% CI 1.44–1.47) globally across 41 countries, with the warming-related increase in OSA prevalence in 2023 estimated to be associated with a loss of approximately 788,000 healthy life years and a workplace productivity loss of $30 billion across 29 countries [25].

Subjectively, older adults consistently reported poorer sleep quality, increased nighttime discomfort, and reduced morning freshness during heat exposure [19,20,26,32]. These effects were more pronounced in elderly, female, and socioeconomically disadvantaged populations, who showed higher vulnerability to heat-related sleep disruption [25,30,43].

Intervention studies indicated that heat-related sleep disruption can be partially mitigated. Cooling strategies such as ventilation, air conditioning use, and environmental modification improved sleep outcomes, including increased sleep efficiency and deeper sleep stages [20,24,26,36]. Laboratory evidence further showed that ventilation at elevated temperatures (e.g., 30 °C conditions) improved deep and REM sleep compared to non-ventilated conditions [28]. Behavioral and educational interventions, including improved access to cooling resources, were associated with modest improvements in total and deep sleep [36].

Overall, while the magnitude effects varied across settings and populations, findings were highly consistent in showing that heat exposure adversely affects both objective and subjective sleep outcomes in older adults. The inclusion of Li et al. (2024) and Lechat et al. (2025) further strengthens this evidence base by demonstrating that heat exposure not only disrupts sleep continuity and duration but also exacerbates sleep-disordered breathing at a population scale, with projected increases in OSA burden under future climate warming scenarios [25,33]. Interventions demonstrated only partial but meaningful mitigation, highlighting the need for scalable, climate-adaptive strategies to protect sleep health in middle-aged and aging populations.

### 3.2. Discussion

This scoping review provides a comprehensive synthesis of evidence examining the relationship between elevated nighttime temperatures, heatwaves, and sleep health in middle-aged and older adults aged 45 and above. Drawing on 31 studies published between 2016 and 2026—spanning six continents, 41 countries, and encompassing over 120 million individuals—our findings demonstrate with remarkable consistency that thermal stress during nocturnal hours represents a clinically meaningful and increasingly urgent threat to sleep health in aging populations. Across diverse methodological frameworks, geographic contexts, and population characteristics, the evidence converges on a coherent picture: as nighttime temperatures rise, older adults sleep less, sleep worse, and recover less effectively. Evidence further indicates that elevated ambient temperatures exacerbate sleep-disordered breathing, with large-scale wearable-based studies demonstrating robust associations between higher temperatures and increased obstructive sleep apnea severity [25,33].

#### 3.2.1. Objective Evidence Across Aging Populations

The burden of heat-related sleep impairment in older adults is well-documented across a wide spectrum of study designs and objective measurement tools. Among the largest and most methodologically rigorous contributions, Lechat et al. [25] analyzed 317,758 individuals across 41 countries using Withings wearable devices and demonstrated that heatwave exposure (≥3 consecutive days above the 90th local temperature percentile) reduced TST by 15–17 min and increased the prevalence of short sleep (<6 h) by 40–43%, with disproportionately stronger effects in older adults. Similarly, Li et al. [31], leveraging 23,197,045 sleep monitoring days from 214,445 Huawei device users across 336 Chinese cities, reported that each 10 °C increase in mean daily temperature increased the odds of sleep insufficiency by 20.1% and reduced TST by 9.67 min, with the most pronounced effects observed in adults aged ≥45 years, women, individuals with BMI ≥ 25, and those residing in high-humidity regions. Minor et al. [29], in a global panel study spanning 68 countries and 7.41 million sleep records, corroborated these findings, showing that nighttime temperatures exceeding 30 °C reduced sleep duration by approximately 14 min, delayed sleep onset, advanced wake time, and increased the probability of insufficient sleep, with elderly individuals, women, and low-income populations bearing the greatest burden and showing no evidence of physiological adaptation over time.

At the national and regional level, Liao et al. [30] utilized 12.5 million Fitbit-tracked sleep days from 14,232 participants in the United States and found that nighttime temperature anomalies reduced TST, worsened sleep efficiency, increased WASO, delayed sleep onset, and diminished REM, deep, and light sleep, with particularly vulnerable subgroups including older adults, females, Hispanics, those with lower SES, and individuals with comorbid cardiovascular disease, depression, or obesity. Obradovich et al. [43], drawing on 766,761 respondents from the US Behavioral Risk Factor Surveillance System (2002–2011), demonstrated that higher nighttime temperature anomalies significantly increased self-reported insufficient sleep, with adults ≥ 65 years showing effect sizes nearly 50% larger than the general population. These large-scale findings are further reinforced by Hajdu [21], whose analysis of 46,446 Hungarian time-use survey respondents revealed that hot days (>25 °C) reduced sleep duration by 5–25 min, with stronger and more consistent reductions in older adults and men.

Cohort-level evidence from Asia further strengthens these conclusions. Zhou et al. [17], using difference-in-difference modeling on CHARLS data from 7240 Chinese adults aged ≥60 years, found that city-level heatwave exposure (≥2–4 consecutive days above the 90th–97.5th temperature percentile) significantly reduced self-reported sleep duration, with the most pronounced effects in women, urban residents, and individuals with chronic diseases. Wang et al. [32], in a fixed-effect analysis of approximately 45,319 Chinese adults across two CFPS survey waves, identified temperatures exceeding 28 °C as a threshold above which the risk of poor sleep increased substantially, with elderly individuals (≥60 years) demonstrating heightened sensitivity attributable to impaired thermoregulatory reserve. Hailemariam et al. [35], analyzing 97,959 observations from the Australian HILDA longitudinal survey (2001–2019), identified an inverted U-shaped relationship between temperature and general health, with hot days associated with reduced wellbeing, though the association with self-reported sleep quality was not robust across all specifications, likely reflecting the limitations of a single-item sleep measure administered only in waves 13 and 17.

Beyond general sleep disruption, elevated ambient temperatures were also associated with worsening of sleep-disordered breathing across two large-scale wearable-based studies. Li et al. (2024) [33], using PPG-based smartwatch monitoring in 51,842 participants with moderate-to-severe OSA risk across 313 Chinese cities (6,232,056 monitoring days), demonstrated a near-linear association between ambient temperature and OSA severity, with each 10 °C increase in daily temperature associated with an 8.4% increase in the odds of OSA exacerbation (95% CI 7.6–9.3%), an increase in AHI of 0.70 events/h (95% CI 0.65–0.76), and a decrease in minimum oxygen saturation of 0.18% (95% CI 0.16–0.19%). These effects were present on the same day only and were significantly stronger in adults aged ≥45 years compared to younger counterparts. Lechat et al. (2025) [25], in a global analysis of 116,620 users of an FDA-cleared under-mattress nearable sensor across 41 countries (~62 million nights), found that high ambient temperatures (99th vs. 25th percentile; 27.3 vs. 6.4 °C) were associated with a 45% higher probability of nightly OSA (RR 1.45, 95% CI 1.44–1.47) and a 49% higher probability of severe OSA (RR 1.49, 95% CI 1.46–1.52), with the warming-related increase in OSA prevalence in 2023 estimated to be associated with a loss of approximately 788,000 healthy life years and a workplace productivity loss of $30 billion across 29 countries. Notably, in contrast to Li et al. (2024) [33], Lechat et al. (2025) [25] found that age did not strongly modify the temperature–OSA association across 10-year age categories, suggesting that, while older adults may be more susceptible to heat-related general sleep disruption, the temperature–OSA relationship may operate more uniformly across age groups when OSA is the primary outcome.

#### 3.2.2. Indoor Thermal Environments and Real-World Monitoring

While population-level studies establish the epidemiological magnitude of heat-related sleep disruption, home-based monitoring studies reveal the granular, real-world mechanisms through which thermal exposure translates into measurable sleep impairment. Baniassadi et al. [37], in a 12-month repeated-measure study of 50 community-dwelling older adults in the Boston metropolitan area (10,903 person-nights), identified a non-linear relationship between bedroom temperature and sleep, with optimal sleep efficiency and TST occurring between 20 and 25 °C, SE declining by approximately 5–10% above 25 °C, and TST falling by ~60 min at 30 °C compared to 22 °C.

The substantial inter-individual variability observed in this study underscores that population-level averages may mask clinically relevant differences in thermal sensitivity among older individuals. Basner et al. [34], monitoring 62 community-based adults over 14 nights in Louisville, Kentucky, found that the highest bedroom temperature quintile was associated with a ~3.4% reduction in sleep efficiency compared to the lowest, with additive impairments from simultaneous exposure to elevated PM2.5, CO_2_, and noise levels—highlighting the compounding role of the built environment in sleep health. Van Loenhout et al. [19] demonstrated in a Dutch cohort of 113 community-dwelling adults aged ≥65 years that indoor bedroom temperatures exceeding 25 °C during summer weeks were a significant predictor of sleep disturbance, with sweating and thirst commonly co-occurring, and with limited access to air conditioning constraining adaptive capacity. Williams et al. [21] provided critical evidence of the role of cooling infrastructure in moderating heat-related sleep disruption, finding that low-income older adults (≥55 years) residing in non-air-conditioned public housing in Cambridge, Massachusetts, experienced significantly worse sleep, elevated heart rate, and increased galvanic skin response compared to those with central AC during a summer heat event (mean indoor temperature 25.6 °C). These findings were corroborated by Milando et al. [42], who demonstrated in a mixed-method field study of 24 low-income urban residents in Boston that personal heat exposure was approximately 3.9 °C higher than weather station measurements and that air conditioning, even when present, did not fully eliminate indoor thermal burden, underscoring the inadequacy of meteorological data alone as a proxy for individual-level heat exposure.

Son et al. [27], studying 18 independent seniors (≥54 years) in Phoenix, Arizona, found that higher nighttime bedroom temperature was paradoxically associated with reduced sleep fragmentation and improved sleep efficiency in a cross-sectional three-day assessment, though it was negatively correlated with overall sleep duration—a finding that may reflect behavioral adaptation or short-term acclimatization in a desert climate population habitually exposed to high temperatures. Uriarte-Otazua et al. [26] added qualitative depth to this evidence base through a high-resolution case study of two older adults living alone in the Basque Country, finding that urban dwelling with indoor nighttime temperatures reaching 28.7 °C was associated with poor sleep, while peri-urban residence with access to natural ventilation and greater mobility supported more favorable nocturnal thermal conditions.

#### 3.2.3. Controlled and Laboratory Evidence

Controlled experimental studies provide mechanistic clarity on the dose–response relationship between bedroom temperature and specific sleep architecture components in older adults. Yan et al. [28], in a carefully controlled PSG study of 16 older adults (≥65 years) in Shanghai, demonstrated that exposure to 30 °C compared to a reference condition of 27 °C reduced TST by 26.3 min, SE by 5.5%, and REM sleep by 5.3 min, while increasing time awake by 27 min. Critically, the addition of ventilation at 30 °C recovered 10.3 min of deep sleep and 3.7 min of REM sleep, providing direct experimental evidence for the restorative value of air movement even in the absence of full cooling. However, experimental evidence establishing precise thermal thresholds for sleep optimization in older adults remains limited, highlighting an important gap in the literature.

Guo et al. [38], in a seasonal observational field study of 197 adults in the Tibet Autonomous Region, found that summer bedroom conditions characterized by elevated temperature, humidity, and CO_2_ significantly impaired sleep efficiency and deep sleep, with CO_2_ identified as the dominant predictor—a finding that has important implications for ventilation design in energy-efficient buildings where air exchange may be limited. In winter, low humidity emerged as the primary driver of sleep disturbance, underscoring the bidirectional nature of environmental thermal stress. Liu et al. [41], in a large retrospective PSG-based study of 5204 adults in Taiwan (mean age 49.5 ± 13.5 years), found that higher long-term ambient temperature was associated with reduced sleep efficiency and elevated WASO, while increased relative humidity was associated with higher AHI, altered sleep staging, and more frequent arousals—a finding of particular relevance given the high prevalence of obstructive sleep apnea in older adult populations, and consistent with the population-level associations between temperature and OSA severity reported by Lechat et al. (2025) and Li et al. (2024) [25,33]. Lappharat et al. [39], in a cross-sectional study of 63 OSA patients in Bangkok, found that higher bedroom temperature was associated with poorer subjective sleep quality (OR 1.46, *p* = 0.044) and that PM10 independently predicted OSA severity, highlighting the co-exposure burden in tropical urban environments.

#### 3.2.4. Seasonal, Geographic, and Longitudinal Patterns

Seasonal and geographic analyses reveal important contextual determinants of thermal sleep vulnerability in older adults. Kume et al. [18], in a prospective comparison of older adults in Japan (n = 37) and Thailand (n = 44) using Actiwatch 2 devices, found that Japanese participants experienced the shortest sleep duration during summer, while Thai participants showed persistently poor and irregular sleep throughout the year, suggesting that chronic tropical heat exposure may engender partial adaptation but does not eliminate sleep impairment. Cepeda et al. [23], in the Rotterdam Study (n = 1166, ≥50 years), found that temperature accounted for 49.4% of seasonal variability in sleep duration among middle-aged adults, with nighttime TST peaking in mid-January, while the oldest-old subgroup showed attenuated seasonal variation—potentially reflecting reduced thermoregulatory responsiveness rather than resilience. Tsuzuki et al. [44], in a four-season repeated-measure actigraphy study of 13 older adults in Japanese elderly care facilities, found that summer indoor nighttime temperatures consistently exceeded 28–29 °C, with men demonstrating worse sleep efficiency and longer wake times than women, and adaptive behaviors such as fan use and window opening providing only partial relief. Li W. et al. [40], in a prospective fixed-effect study of 98 adults with episodic migraine in the Greater Boston area (4406 sleep nights), found that higher ambient temperature independently predicted increased WASO and reduced sleep efficiency, while elevated ozone was associated with longer sleep duration findings that point to complex multi-pollutant interactions in the urban sleep environment that remain incompletely characterized. At a global scale, Lechat et al. (2025) demonstrated that the effect size of temperature on nightly OSA probability varied substantially by country and was generally strongest in European nations, with up to a ~2-fold increase in OSA probability at the 99th temperature percentile, highlighting that geographic and climatic context meaningfully moderates the temperature–sleep-disordered breathing relationship [25].

#### 3.2.5. Interventions and Protective Factors

The evidence consistently supports ventilation and cooling as effective, if incompletely accessible, countermeasures to heat-related sleep impairment in older adults. Huang et al. [36], in a rural elderly Chinese intervention study (n = 41), demonstrated that targeted education, financial subsidies, and cooling provision mitigated heat-related TST reduction and improved deep sleep duration. Yan et al. [28] provided experimental confirmation that ventilation at 30 °C partially recovered the deep and REM sleep lost relative to a 27 °C condition. Cheong and Gaynanova [24] found that frequent air conditioning use attenuated the 2% per-degree-Celsius reduction in sleep efficiency observed in non-AC users. Collectively, these findings highlight the public health imperative of ensuring equitable access to cooling infrastructure, particularly among low-income, elderly, and urban populations who face the greatest thermal burden and the least adaptive capacity [20,26,42]. The potential protective role of cooling extends beyond general sleep outcomes to sleep-disordered breathing: given the consistent evidence that higher ambient temperatures exacerbate OSA severity [25,33], maintaining comfortable indoor temperatures on high-temperature nights may represent an actionable strategy for OSA management in older adults, warranting evaluation in future intervention studies.

#### 3.2.6. Physiological Mechanisms

The convergent epidemiological and experimental evidence reviewed here is mechanistically coherent with established physiology of thermoregulation and sleep. Nocturnal heat exposure impairs the normal decline in core body temperature required for sleep onset and maintenance, a process coordinated by the preoptic area (POA) of the hypothalamus through GABAergic–glutamatergic circuits, pituitary adenylate cyclase-activating polypeptide (PACAP), and brain-derived neurotrophic factor (BDNF) [46,47,48]. Aging progressively attenuates this thermoregulatory machinery—reducing sweat gland responsiveness, peripheral vasodilation capacity, and hypothalamic thermal sensitivity—thereby narrowing the thermal window within which restorative sleep can occur. Elevated nighttime temperatures can further suppress melatonin secretion and disrupt circadian timing [49], amplifying sleep fragmentation and reducing NREM and REM sleep stages. At the cellular level, sustained thermal stress increases mitochondrial reactive oxygen species production, promotes oxidative damage [50], and dysregulates circadian gene expression [51], with downstream consequences for metabolic, inflammatory, and neuronal pathways. High temperatures may also exacerbate upper airway instability during sleep by reducing upper airway dilator muscle activity, increasing pharyngeal collapsibility, and promoting airway inflammation—mechanisms consistent with the temperature-related increases in AHI and OSA exacerbation observed by Li et al. (2024) and Lechat et al. (2025), and mechanistically aligned with the broader literature on OSA pathophysiology in aging populations [25,33]. Heat-related sleep disruption may additionally compromise nocturnal glymphatic clearance of neurotoxic proteins including β-amyloid and tau [52]—a mechanism of particular concern given the association between heatwave exposure and cognitive impairment risk observed by Zhou et al. [16]—though direct causal evidence in humans remains to be established. Elevated sympathetic activity and altered cardiovascular responses during nocturnal heat exposure further compound these risks [53], particularly in older adults with pre-existing cardiovascular vulnerability.

#### 3.2.7. Disparities and Future Directions

A consistent cross-cutting finding across this review is that heat-related sleep impairment is not uniformly distributed. Women, older adults, individuals with lower socioeconomic status, residents of urban heat islands, those with chronic disease, and populations in lower-GDP countries and tropical climates bear disproportionately greater burdens, as demonstrated across multiple large-scale studies [29,30,32,43]. These inequalities are likely to intensify under projected climate warming scenarios: Minor et al. [29] estimated that climate change could increase annual sleep loss by 50–58 h per year by 2099 under high-emission pathways, while Liao et al. [30] projected 8.5–24 additional hours of annual sleep loss per person by 2100 depending on climate zone and emission scenario. The societal burden of heat-related sleep-disordered breathing is similarly projected to escalate substantially: Lechat et al. (2025) estimated that scenarios with projected temperatures ≥ 1.8 °C above pre-industrial levels could incur a 1.2- to 3-fold increase in OSA burden by 2100, with an associated economic cost of 0.9 to 2.0 trillion USD in labor loss over the 2023–2100 period, underscoring that the consequences of heat-related sleep disruption extend well beyond general sleep insufficiency to encompass clinically significant sleep-disordered breathing at a population scale [25]. These projections underscore the urgency of integrating sleep health into climate adaptation frameworks, urban planning, and public health policy targeting aging populations. Notably, it is increasingly recognized that disparities in sleep health across gender, socioeconomic status, and geographic region are likely amplified by differential heat stress vulnerability, reinforcing the need to consider environmental heat as a structural determinant of sleep equity in middle-aged and aging populations [54].

Future research should prioritize polysomnography-validated wearable assessments in ecologically valid settings, longitudinal designs with repeated objective thermal and sleep measurements, and inclusion of diverse older adult populations across socioeconomic, geographic, and clinical strata. Standardization of heatwave definitions, indoor versus outdoor temperature measurement, and sleep outcome reporting would substantially enhance cross-study comparability. Age-stratified analyses of temperature effects on sleep-disordered breathing outcomes, including OSA severity and oxygen desaturation, are particularly warranted given the inconsistency between Li et al. (2024), who found stronger effects in adults aged ≥45 years, and Lechat et al. (2025), who found no strong age modification, and given the established age-related increase in OSA prevalence and upper airway vulnerability in older populations [25,33]. Intervention research evaluating scalable, low-cost cooling strategies—including passive ventilation, green infrastructure, and targeted subsidies—is urgently needed to translate epidemiological evidence into actionable public health responses.

#### 3.2.8. Limitations

Several limitations of this scoping review warrant consideration. The methodological constraints inherent to scoping review design preclude formal risk-of-bias appraisal and meta-analytic synthesis; consequently, the evidence presented here reflects a systematic mapping of the available literature rather than a weighted quantitative estimate of effect magnitude, and causal inference should be approached with appropriate circumspection.

The literature search was confined to two electronic databases (PubMed and Scopus), and gray literature, institutional reports, conference abstracts, and non-English language publications may have been insufficiently captured. Consequently, some potentially relevant reports, dissertations, conference proceedings, or non-indexed studies may not have been captured. Although no explicit language restriction was applied during screening, the predominance of the English-language indexed literature introduces a potential language and publication bias, with studies reporting null or non-significant findings less likely to have been identified.

Substantial methodological heterogeneity across included studies constitutes a further limitation to cross-study synthesis. Heat exposure was operationalized using widely divergent approaches—ranging from percentile-based heatwave indices and absolute meteorological thresholds to indoor sensor-based measurements and individualized wearable-derived estimates—precluding direct comparability of exposure definitions. Similarly, sleep outcomes were assessed using a broad spectrum of instruments, including polysomnography, actigraphy, wearable consumer devices, under-mattress sensors, and self-reported questionnaires, each with distinct psychometric properties, measurement precision, and susceptibility to bias. The absence of standardized case definitions for both heat exposure and sleep impairment across the field represents a structural limitation that extends beyond this review and substantially constrains the interpretability of pooled findings.

Although this review was designed to focus on adults aged 45 years and older, a proportion of included studies also included a younger population. To limit conclusions from these studies to our target group, only age-stratified results from these studies were taken into account. Studies including older adults, but without subgroup analysis, were excluded. This limitation is particularly relevant to some of the OSA studies. Furthermore, institutionalized older adults, nursing home residents, and individuals with significant functional impairment or cognitive decline were systematically underrepresented across the included literature, potentially resulting in underestimation of heat-related sleep vulnerability in the most clinically at-risk segments of the aging population.

The geographic distribution of included studies, while spanning six continents, was disproportionately weighted toward high-income settings—particularly China, the United States, and Western Europe—with limited representation from Sub-Saharan Africa, South and Southeast Asia, and Latin America. Given that these regions are projected to experience the most severe increases in heat exposure under future climate scenarios, and that aging populations in low- and middle-income countries face compounded vulnerabilities related to inadequate cooling infrastructure and limited healthcare access, the generalizability of current findings to these settings remains uncertain.

Finally, the cross-sectional or short-term longitudinal design of the majority of included studies limits insight into the long-term consequences of repeated or chronic heat-related sleep disruption, including cumulative effects on cognitive decline, cardiometabolic health, and mortality risk in older adults—outcomes of considerable clinical and public health importance that remain insufficiently characterized in the existing literature.

## 4. Conclusions

This scoping review shows that elevated nighttime temperatures are a growing threat to sleep health in middle-aged and older adults aged 45 and above. Heat consistently reduces sleep duration, efficiency, and restorative stages, with middle-aged and older adults being especially vulnerable due to progressive thermoregulatory decline. Elevated ambient temperatures are increasingly associated with worsening obstructive sleep apnea severity. Large-scale wearable-based studies consistently report increased AHI, higher odds of OSA exacerbation, and reduced minimum oxygen saturation under higher temperature exposure. Modeling projections further suggest that, under continued global warming scenarios, the burden of sleep-disordered breathing may substantially increase at the population level by the end of the century. Environmental, housing, and socioeconomic factors further influence susceptibility. Despite accumulating evidence, gaps remain in standardized exposure metrics, objective sleep assessments, and focus on older populations. Future research should combine environmental monitoring, sleep physiology, and gerontology to develop targeted interventions. Public health strategies, including housing adaptation, behavioral cooling, and equitable access to cooling technologies, are needed to protect sleep and wellbeing as extreme heat events increase.

## Data Availability

All data of this scoping review are presented in the manuscript in Table 1.
